# Decreased serum concentrations of sphingosine-1-phosphate in sepsis

**DOI:** 10.1186/s13054-015-1089-0

**Published:** 2015-10-26

**Authors:** Martin Sebastian Winkler, Axel Nierhaus, Maximilian Holzmann, Eileen Mudersbach, Antonia Bauer, Linda Robbe, Corinne Zahrte, Maria Geffken, Sven Peine, Edzard Schwedhelm, Guenter Daum, Stefan Kluge, Christian Zoellner

**Affiliations:** Department of Anaesthesiology, University Medical Center Hamburg-Eppendorf, Martinistr. 52, 20246 Hamburg, Germany; Department of Intensive Care Medicine, University Medical Center Hamburg-Eppendorf, Martinistr. 52, 20246 Hamburg, Germany; Institute of Clinical Pharmacology and Toxicology, University Medical Center Hamburg-Eppendorf, Martinistr. 52, 20246 Hamburg, Germany; Institute of Transfusion Medicine, University Medical Center Hamburg-Eppendorf, Martinistr. 52, 20246 Hamburg, Germany; Clinic and Polyclinic for Vascular Medicine, University Heart Center, Martinistr. 52, 20246 Hamburg, Germany

## Abstract

**Introduction:**

Sphingosine-1-phosphate (S1P) is a signaling lipid that regulates pathophysiological processes involved in sepsis progression, including endothelial permeability, cytokine release, and vascular tone. The aim of this study was to investigate whether serum-S1P concentrations are associated with disease severity in patients with sepsis.

**Methods:**

This single-center prospective-observational study includes 100 patients with systemic inflammatory response syndrome (SIRS) plus infection (n = 40), severe sepsis (n = 30), or septic shock (n = 30) and 214 healthy blood donors as controls. Serum-S1P was measured by mass spectrometry. Blood parameters, including C-reactive protein (CRP), procalcitonin (PCT), interleukin-6 (IL-6), lactate, and white blood cells (WBCs), were determined by routine assays. The Sequential Organ Failure Assessment (SOFA) score was generated and used to evaluate disease severity.

**Results:**

Serum-S1P concentrations were lower in patients than in controls (*P* < 0.01), and the greatest difference was between the control and the septic shock groups (*P* < 0.01). Serum-S1P levels were inversely correlated with disease severity as determined by the SOFA score (*P* < 0.01) as well as with IL-6, PCT, CRP, creatinine, lactate, and fluid balance. A receiver operating characteristic analysis for the presence or absence of septic shock revealed equally high sensitivity and specificity for S1P compared with the SOFA score. In a multivariate logistic regression model calculated for prediction of septic shock, S1P emerged as the strongest predictor (*P* < 0.001).

**Conclusions:**

In patients with sepsis, serum-S1P levels are dramatically decreased and are inversely associated with disease severity. Since S1P is a potent regulator of endothelial integrity, low S1P levels may contribute to capillary leakage, impaired tissue perfusion, and organ failure in sepsis.

## Introduction

Sepsis is a major clinical and health-care problem affecting millions of people worldwide [1]. In the US, hospitalizations for sepsis doubled from 326,000 in 2000 to 727,000 in 2008 [2]. Despite considerable efforts to understand the pathophysiology of sepsis and to find new treatments over the last decades, mortality remains high. In 2008, in the US, the mortality rate of patients with septicemia or sepsis was 17 % whereas the mortality rate of other hospitalizations was only 2 % [2]. Immediate therapy of sepsis is of great importance as patient survival depends on timely antibiotic treatment [3], which should be considered already in the pre-hospital setting [4].

Sepsis is an acute life-threatening condition of the host to an underlying infection. With increasing severity, the effectors of the immune response are damaging tissues and organs [5]. Importantly, pro-inflammatory and anti-inflammatory responses coexist in sepsis and invariably cause severe organ dysfunction, septic shock, and immunosuppression [6].

Hallmarks for sepsis severity include endothelial barrier disruption, uncontrolled cytokine secretion, and hypotension, all of which are potentially regulated by sphingosine-1-phosphate (S1P).

S1P is a bioactive sphingolipid that regulates many physiological as well as pathophysiological processes in the vasculature and immune systems [7, 8]. S1P is present at high concentrations in plasma where it is bound mainly to albumin or high-density lipoprotein (HDL). It is produced by two sphingosine-kinases (SphK 1 and 2) and degraded by dephosphorylation or cleavage. Most of its effects are mediated by five specific G protein-coupled receptors (S1PR1-5). S1PR1 is critical for endothelial integrity as has been demonstrated by the lethality of genetic S1PR1 deletion in mice, which die *in utero* from hemorrhage [9]. Moreover, mice with greatly decreased plasma-S1P levels display increased vascular leakage and impaired survival after inflammatory challenge [10]. In contrast, intravenous application of S1P to mice and dogs attenuates barrier dysfunction in lungs following lung injury [11]. Anti-inflammatory properties of S1P have been observed in a mouse H1N1-influenza virus infection model, in which oral administration of S1PR1 agonists inhibits early pro-inflammatory cytokine production and innate immune cell recruitment [12]. At last, a role for S1P to maintain the vascular tone has been demonstrated in various arterial beds [13]. Considering these observations, we sought to investigate whether serum-S1P levels are altered in sepsis and whether they are associated with sepsis severity.

## Methods

### Study population

From March until December 2014, 100 patients (>18 years old) who were admitted to the intensive care unit (ICU) of the University Medical Center Hamburg-Eppendorf (Hamburg, Germany) with different stages of sepsis were enrolled after informed consent had been obtained from patients or their legal representatives. The study protocol was approved by the ethical review committee of the local medical chamber of Hamburg (Aerztekammer Hamburg, reference number PV4550).

Inclusion criteria were a diagnosed infection or a clinical syndrome pathognomonic for an infection. Patients were categorized into three groups on the basis of the criteria published by the American College of Chest Physicians/Society of Critical Care Medicine [14]. Systemic inflammatory response syndrome (SIRS) plus infection/sepsis (group A) was defined as an infectious disease with at least two SIRS criteria: temperature of more than 38 °C or less than 36 °C, heart rate of more than 90 beats per minute, respiratory rate of more than 20 per minute or partial pressure of arterial carbon dioxide (PaCO_2_) of less than 32 mm Hg, and white blood cell (WBC) count of more than 12 × 10^9^/l or less than 4 × 10^9^/l. Severe sepsis (group B) was defined as sepsis and the presence of the following organ failure: acute encephalopathy, thrombocytopenia (reduction of at least 30 % in 24 hours or platelets of not more than 100 × 10^9^/l), hypoxia (partial pressure arterial oxygen (PaO_2_) of not more than 4.3 kPa/75 mm Hg with room air or PaO_2_/fraction of inspired oxygen (FiO_2_) of not more than 33 kPa/250 mm Hg under oxygen), renal dysfunction (urine output of not more than 0.5 ml/kg per hour in 2 hours despite sufficient fluids), resuscitation or increase of serum creatinine (of more than 2×) or both, metabolic acidosis (Base excess of not more than −5 mmol/l or lactate of more than 1.5 mmol/l), or hypotension/cardiovascular dysfunction (after fluid resuscitation with at least 30 ml/kg of crystalloid). Septic shock (group C) was defined as severe sepsis with hypotension requiring vasopressor support or persistent hypotension for more than 1 hour despite adequate fluid resuscitation.

The control cohort consisted of 214 volunteers donating blood at the Institute of Transfusion Medicine, University Medical Center Hamburg-Eppendorf (Hamburg, Germany). All blood donations were performed in accordance with the latest (2010) guidelines of the German Federal Medical Council (Bundesaerztekammer), which specifically exclude blood donations from subjects with severe health problems. In accordance with ethics regulations, samples were anonymized; only age and gender were reported. Blood was processed as described below for patients.

### Clinical evaluations and assays

For all patients with diagnosed sepsis, a Simplified Acute Physiology Score (SAPS) II [15] and a Sequential Organ Failure Assessment (SOFA) score were generated [16]. The SOFA score was calculated on admission as previously described [16]. Within the first 24 hours after inclusion, the fluid balance (calculated as fluid intake minus fluid loss) was recorded and blood was drawn to measure S1P and to determine clinical parameters, including WBC counts, creatinine, lactate, C-reactive protein (CRP), procalcitonin (PCT), and interleukin-6 (IL-6). All routine clinical assays were performed at the Department of Clinical Chemistry at the University Hospital Hamburg-Eppendorf.

### Serum preparation and S1P measurements

Blood samples from controls and patients were processed the same way. After coagulation at 4 °C, samples were cleared by centrifugation and serum immediately frozen and stored at −80 °C until S1P was measured. Serum-S1P measurements were performed by using a previously described protocol with minor modifications [17]. After the addition of internal standard (1 nmol/ml C17-S1P; Avanti Polar Lipids, Alabaster, AL, USA), serum was de-proteinated by the addition of acetonitrile (final concentration of 70 %). Extracts were cleared by centrifugation and subjected to reverse-phase chromatography on a Zorbax SB-C8 column (2.1 × 50 mm; Agilent Technologies, Santa Clara, CA, USA) at a flow rate of 0.35 ml/min. S1P was eluted by a binary gradient (2.5 % methanol, 2.5 % acetonitrile, 0.1 % formic acid to 30 % methanol, 30 % acetonitrile, 0.1 % formic acid; % = volume %) and measured by a Varian MS 1200 mass spectrometer by using multiple reaction mode in which the M + H S1P parent ion (m/z = 380) is fragmented to form a daughter ion at m/z = 264, which is then used for quantitation. The internal standard (C17-S1P) with the m/z 366-to-250 transition was used to correct for variations in sample preparation and instrument response. A calibration curve (0.1-3 nmol/ml S1P) was generated to calculate absolute S1P concentrations in samples.

### Statistical analysis

The primary variable is the serum-S1P concentration in nanomoles per liter. All variables were tested for normal distribution and transformed if necessary as indicated in the figures or tables. Differences between groups were tested for significance by using either the non-parametric Mann–Whitney *U* test for two groups or the Kruskal-Wallis analysis of variance for more than two groups. Data are presented as median with interquartile range or as mean with standard error of the mean. Correlations were calculated by using linear regression or the Spearman test. Receiver operating characteristic (ROC) curves were generated, and areas under the curve (AUC) were calculated. Odds ratios with 95 % confidence intervals (CIs) were computed by using a multivariate logistic regression model with septic shock as the dependent variable. For all tests, a *P* value of less than 0.05 was considered significant. Statistical analyses were performed by using SPSS (version 21; IBM Corporation, Armonk, NY, USA) with guidance from members of the Department of Medical Biometry and Epidemiology at the University Hospital Hamburg-Eppendorf.

## Results and discussion

### Patients with sepsis have lower serum-S1P concentrations than controls

**Fig. 1:**
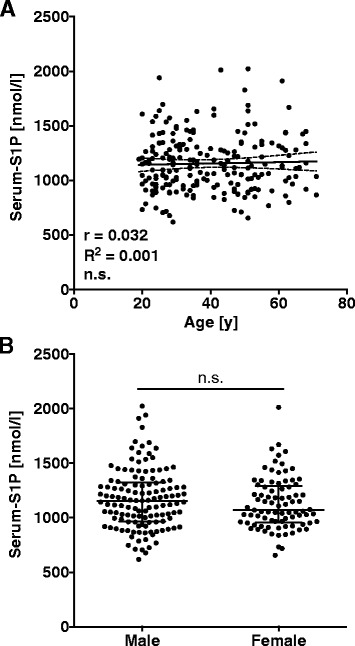
Serum-S1P is not dependent on age (**a**) or gender (**b**) in healthy controls. Linear regression analysis with 95 % confidence interval (**a**) and median with interquartile range are given and groups were compared using Mann–Whitney *U* test (**b**). *n.s.* non-significant, *S1P* sphingosine-1-phosphate

**Fig. 2:**
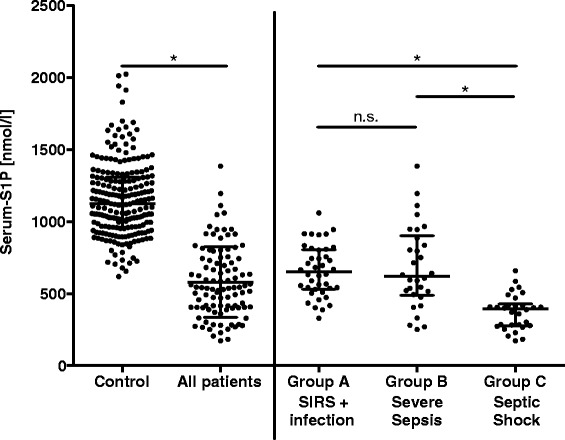
Serum-S1P concentrations in controls and sepsis patients. Bars indicate the median and interquartile range. Mann–Whitney *U* test was used to compare controls versus the entire patient cohort (All patients), and analysis-of-variance Kruskal-Wallis test was used for comparisons between the three patient groups (*P <0.01). *n.s.* non-significant, *S1P* sphingosine-1-phosphate, *SIRS* systemic inflammatory response syndrome

Serum-S1P concentrations were measured in 100 patients with sepsis and 214 healthy controls by mass spectrometry after de-proteinization of serum with acetonitrile. This method determines the total S1P content in serum regardless of the nature of the S1P-binding protein. It should be noted that, during coagulation, platelets release S1P and so contribute to total serum-S1P levels [18]. The two study groups did not differ regarding sex distribution; however, the control group was younger on average (Table 1). Neither an age dependency nor a gender bias was detected in controls (Fig. 1). Patients with sepsis had significantly lower mean serum-S1P levels (580 ± 24 nmol/l) than controls (1156 ± 17 nmol/l; Fig. 2). The patient cohort was then divided into three groups (groups A-C) of increasing disease severity. Consistent with this classification, ICU stays were the longest for patients with septic shock (group C), followed by patients with severe sepsis (group B) or sepsis (group A). Also, the death rate in groups B and C was about 10 times higher than in group A (Table 1). The three patient groups did not differ in age or sex distribution or in reasons for admission (medical or surgical; Table 1). When serum-S1P levels between the three groups were compared, group C presented with significantly lower levels than group A or B (Fig. 2).

**Table 1:** Characteristics of study groups

	Controls	All patients	Group A	Group B	Group C	**Significance (P) – Group A to C
			SIRS + Infection	Severe sepsis	Septic shock	
Number	214	100	40	30	30	N/A
*Age, years	36 (27–51)	59 (51–70)	59 (46–67)	60 (53–72)	65 (56–74)	n.s.
Men/Women, n (%)	129/85 (60/40)	58/42 (58/42)	22/18 (55/45)	20/10 (67/33)	16/14 (53/47)	n.s.
Admission medical/surgical, n (%)	N/A	56/44 (56/44)	20/20 (50/50)	20/10 (67/33)	16/14 (53/47)	n.s.
*SAPS II, median (IQR)	N/A	33 (24–42)	28 (17–35)	34 (30–41)	42 (30–47)	<0.01
*Length of ICU stay, days	N/A	7 (2–11)	2 (1–6)	8 (4–12)	11 (7–21)	<0.01
Death on ICU, n (%)	N/A	15 (15.0)	1 (2.5)	7 (23.3)	7 (23.3)	<0.05

*SIRS* systemic inflammatory response syndrome, *N/A* not applicable, *n.s.* non-significant, *SAPS II* Simplified Acute Physiology Score II, *IQR* interquartile range, *ICU* intensive care unit

*Data are presented as median and IQR

**Groups were compared by using analysis-of-variance Kruskal-Wallis test

### There is an association between serum-S1P concentrations and the Sequential Organ Failure Assessment score

**Fig. 3:**
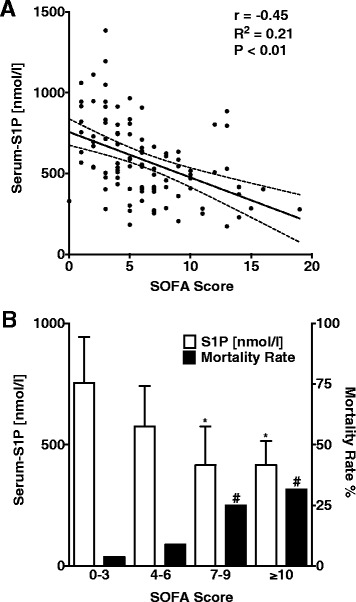
In patients with sepsis, serum S1P is associated with SOFA score and mortality. **a** Linear regression analysis and 95 % confidence interval with SOFA score as the dependent variable and serum-S1P concentrations. **b** Mortality rate in relation to the serum-S1P concentration and the SOFA score. Bars indicate medians and interquartile range for S1P. *Analysis-of-variance Kruskal-Wallis test (*P* <0.01). ^#^Chi-square test (*P* <0.05). *S1P* sphingosine-1-phosphate, *SOFA* Sequential Organ Failure Assessment

SOFA scores indicate disease severity in critically ill patients regardless of the underlying disease and therefore are generally used to predict mortality in ICU patients [16]. Linear regression analysis revealed a strong inverse correlation between SOFA scores and serum-S1P levels with a regression coefficient (r) of −0.45 (*P* < 0.01; Fig. 3a). To demonstrate that serum-S1P is linked not only to disease severity but also to mortality, patients were classified into four groups with increasing SOFA score. For each group, the mortality rate and average serum-S1P concentrations were calculated. We also found that mortality was inversely associated with serum-S1P levels (Fig. 3b).

### Serum-S1P concentrations predict septic shock with high accuracy

**Fig. 4:**
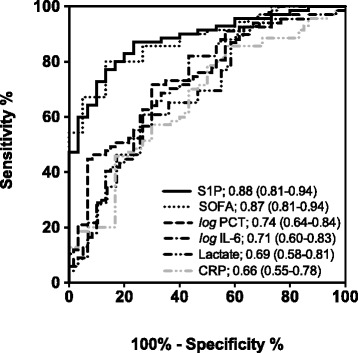
Receiver operating characteristic (ROC) curves for the prediction of septic shock. PCT and IL-6 have been transformed to log_10_ scale to achieve normal distribution. *AUC* area under the curve (95 % confidence interval), *CRP* C-reactive protein, *IL-6* interleukin-6, *PCT* procalcitonin, *S1P* sphingosine-1-phosphate, *SOFA* Sequential Organ Failure Assessment score

Given the association found between the SOFA score and serum-S1P, we also tested for correlations between S1P and the common inflammatory markers such as IL-6, PCT, and CRP as well as for creatinine, lactate, and fluid balance, all of which are increased in sepsis. All parameters tested showed a statistically significant inverse correlation to S1P with the strongest determined for PCT, followed by IL-6, CRP, creatinine, lactate, and fluid balance (Table 2). To compare the potential of various parameters with S1P as to indicate septic shock within patients with sepsis, an ROC analysis, including S1P, the SOFA score, IL-6, CRP, PCT, and lactate, was performed. Among all parameters tested, SOFA score and S1P emerged as the most powerful indicators of septic shock, with almost identical AUCs of 0.88 (95 % CI, 0.82-0.95) for S1P and 0.87 (95 % CI 0.81-0.95) for SOFA score (Fig. 4). The ROC curves for all other markers were similar, with AUCs of between 0.66 and 0.74. To further demonstrate the potential of SOFA score and S1P to predict septic shock, a multivariate logistic regression analysis for all parameters was performed. Multivariate logistic regression showed S1P as the strongest predictor (*P* < 0.001) followed by SOFA score (*P* <0.01) and all other parameters (Table 3).

**Table 2:** Correlations between serum-S1P concentrations and various clinical parameters

	Spearman rho	Significance (*P* value)
PCT	−0.52	<0.01
IL-6	−0.39	<0.01
CRP	−0.28	<0.01
Creatinine	−0.25	<0.05
Lactate	−0.24	<0.05
Fluid balance	−0.23	<0.05

*S1P* sphingosine-1-phosphate, *PCT* procalcitonin, *IL-6* interleukin-6, *CRP* C-reactive protein

Correlations between S1P and PCT, IL-6, CRP, creatinine, lactate, and fluid balance were assessed by using the Spearman rank test

**Table 3:** Multivariate-logistic regression of S1P, SOFA score, PCT, IL-6 CRP, and WBC as predictors of septic shock (dependent variable)

Variable	Regression coefficient	Odds ratio (95 % CI)	Significance (*P* value)
S1P, nmol/l	−0.012	0.988 (0.982-0.995)	<0.001
SOFA score, points	+0.481	1.618 (1.146-2.284)	<0.01
^a^ *log* PCT, μg/l	−0.376	0.686 (0.205-2.299)	n.s.
^a^ *log* IL-6, ng/l	+0.118	1.126 (0.297-4.262)	n.s.
CRP, mg/l	+0.006	1.006 (0.997-1.016)	n.s.
WBC count, ×10^9^/l	−0.007	0.993 (0.888-1.110)	n.s.
Lactate, nmol/l	+0.511	1.667 (0.900-3.089)	n.s.

*S1P* sphingosine-1-phosphate, *SOFA* Sequential Organ Failure Assessment, *PCT* procalcitonin, *IL-6* interleukin-6, *CRP* C-reactive protein, *WBC* white blood cell, *CI* confidence interval, *n.s.* non-significant

^a^PCT and IL-6 have been transformed to log_10_ scale to achieve normal distribution

### Discussion

The main findings of our study are that patients with sepsis, and particularly those with hypotensive septic shock, have decreased serum S1P concentrations and that high serum S1P levels might be able to rule out the risk of having septic shock with a high probability. Serum-S1P concentrations are associated with sepsis severity.

Our serum-S1P measurements in controls are in agreement with two previous studies that reported average serum-S1P concentrations for healthy control groups of 900 and 1400 nmol/l [19, 20]. In patients with sepsis, serum-S1P is approximately 50 % lower than in controls (Fig. 2). Moreover, serum-S1P concentrations were lowest in the most severely affected patients (group C, septic shock; Fig. 2); and a multivariate regression analysis with septic shock as the dependent variable showed that a small decrease in serum-S1P concentration of only 1 nmol/l was associated with an increased risk of developing septic shock of 1.2 % (Table 3).

As blood samples from patients and controls were handled and processed the same way and as we did not observe an age dependency or gender bias for serum-S1P concentrations, we conclude that the difference in serum-S1P concentrations between patients and controls was due to sepsis.

Sepsis is characterized by multiple pathophysiological processes, including immunological stimulation, systemic inflammation, and coagulopathy, which vary in degree from patient to patient [21, 22]. Therefore, one might expect that a complex score addressing diverse clinical parameters is required to assess sepsis severity. For instance, the SOFA score that predicts progression and mortality of critically ill patients with high accuracy consists of evaluations of six different organ systems: the respiratory, cardiovascular, hepatic, renal, neurological, and coagulation systems [16]. It seems remarkable that the power of an individual parameter, S1P, to predict septic shock is comparable to that of the complex SOFA score. An explanation for this may be that S1P inhibits multiple processes relating to sepsis severity:

1. Endothelial barrier disruption plays a critical role in sepsis pertaining to vascular leakage, organ failure, and septic shock. Direct involvement of plasma-S1P in regulating barrier function has been demonstrated in an inflammation lung injury model in mice, where plasma-S1P levels had been lowered by knocking out sphingosine-kinase-1 and −2 alleles. After application of leak-inducing agents such as histamine or platelet-activating factor, mice with decreased plasma-S1P show increased leakage and decreased survival [10]. Conversely, increasing plasma-S1P concentrations by intravenous administration of S1P to lung-injured mice or dogs decreased leakage and edema formation [11, 23].
2. The hyper-inflammatory phase in sepsis is characterized by excessive release of various cytokines [24, 25]. S1P has pro- as well as anti-inflammatory effects [8, 26]. In mice infected with the H1N1 influenza virus, oral or intratracheal administration of S1PR1 agonists inhibits cytokine release and leukocyte recruitment by pulmonary endothelial cells [12]. This anti-inflammatory mechanism may be diminished in sepsis patients with low serum-S1P. An important pro-inflammatory role for S1P in inflammation is the regulation of lymphocyte egress from lymphatic organs into the blood. This process depends on S1PR1, which is used by lymphocytes to detect the S1P gradient between tissue and blood [27]. Interestingly, decreased S1P in patients with sepsis might contribute to the hypo-inflammatory phase of the disease, which is characterized by immune-paralysis with concomitant lymphopenia and typically follows the hyper-inflammatory phase [21]. Although lymphocyte apoptosis has been suggested as the main cause for lymphopenia in sepsis [28], we cannot exclude the possibility that other mechanisms such as altered S1P-dependent lymphocyte egress also contribute.
3. Low S1P levels in patients with sepsis may also contribute to hypotension in septic shock since S1P regulates vascular tone. Intravenous infusion of S1P into anesthetized rats results in decreased blood flow in mesenteric and renal arteries because of vasoconstriction [29]. In rat mesenteric microvessels, S1P increases vasoconstriction in vessels that had been maximally pre-constricted with alpha-adrenergic agonists [30]. This observation suggests that S1P might be used in septic shock when patients no longer respond to catecholamines. Moreover, overexpression of sphingosine-kinase-1 in mice has been found to increase vascular tone; conversely, overexpression of the S1P degrading S1P-phosphoyhdrolase reduced vascular tone [31, 32].

Although taken together these observations encourage the development of interventional studies to test the possibility that elevating S1P levels may be beneficial for patients with sepsis, it should be taken into account that S1P effects depend on the expression pattern of S1P receptors. It is likely that activation of endothelial S1PR1 antagonizes endothelial permeability [33], but activation of S1PR2 may have the opposite effect [34]. Moreover, S1P may have different functions in the various stages of sepsis [34]. Therefore, activating or blocking specific S1P receptors may be a more promising approach to alleviate sepsis symptoms than simply increasing S1P blood concentrations.

### Regulation of serum-S1P concentrations

It is unclear exactly how serum-S1P concentrations are regulated in healthy people, and we can only speculate why they decrease in sepsis. As both platelets and red blood cells store and release S1P [35–37], thrombocytopenia and anemia in patients with sepsis may contribute to their low serum-S1P concentrations [38, 39]. Although data from bone marrow transplant experiments between wild-type and inducible sphingosine-kinase knockout mice suggest a critical role for hematopoietic cells to determine plasma-S1P levels [36], observations that anemic or thrombocytopenic mice have normal plasma S1P levels suggest additional sources for S1P. One strong candidate is the endothelium, as endothelial cells produce S1P in response to laminar flow [40]. Serum-S1P levels may also depend on the concentration of S1P carrier proteins. About 50 % of S1P in plasma is bound to high-density lipoprotein HDL [41], and apolipoprotein M (ApoM) was recently identified as the responsible binding protein [42]. In sepsis, both HDL and ApoM concentrations are decreased, and like S1P, they are negatively associated with sepsis severity [43–45].

Taken together, decreased endothelial S1P production and loss of S1P sources and carrier proteins may all contribute to decreased S1P levels in patients with sepsis.

Decreased serum-S1P concentrations have also been reported in patients with dengue fever. As in sepsis, a hallmark of this disease is the loss of endothelial barrier function which is maintained by S1P/S1PR1 signaling [20]. It is an interesting possibility that, in these diseases, endothelial damage may cause decreased S1P production, which in turn may further promote endothelial damage. If so, elevating S1P levels may break this vicious cycle and constitute a novel approach to stabilizing patients who have vascular leakage.

Limitations of our study are that it was carried out at a single center and involved relatively small numbers of patients with sepsis. Nevertheless, we believe that our observations warrant follow-up studies with larger patient groups to confirm the power of serum-S1P to predict sepsis severity as well as studies to investigate whether patients with sepsis might benefit from therapeutically elevating serum-S1P levels.

## Conclusion

Our observations of low concentrations of S1P in sepsis and septic shock suggest that S1P might play a decisive pathophysiological role in sepsis by regulating endothelial permeability, cytokine release, and vascular tone.

## Key messages

- Concentration of serum sphingosine-1-phosphate (S1P) is decreased in patients with sepsis.
- Decreased serum-S1P concentrations are associated with sepsis severity.

## Abbreviations

ApoM: Apolipoprotein M
AUC: Area under the curve
CI: Confidence interval
CRP: C-reactive protein
HDL: High-density lipoprotein
ICU: Intensive care unit
IL-6: Interleukin-6
PaO_2_: Partial pressure arterial oxygen
PCT: Procalcitonin
ROC: Receiver operating characteristic
S1P: Sphingosine-1-phosphate
S1PR: Sphingosine-1-phosphate receptor
SIRS: Systemic Inflammatory Response Syndrome
SOFA: Sequential Organ Failure Assessment score
WBC: White blood cell
